# Differential gene expression analysis in blood of first episode psychosis patients

**DOI:** 10.1016/j.schres.2019.05.011

**Published:** 2019-07

**Authors:** Daniel J. Leirer, Conrad O. Iyegbe, Marta Di Forti, Hamel Patel, Elena Carra, Sara Fraietta, Marco Colizzi, Valeria Mondelli, Diego Quattrone, John Lally, Olesya Ajnakina, Sang Hyuck Lee, Charles J. Curtis, Gerome Breen, Carmine Pariante, Katherine Aitchison, Paola Dazzan, Robin M. Murray, Richard J.B. Dobson, Stephen J. Newhouse

**Affiliations:** a MRC Social, Genetic and Developmental Psychiatry (SGDP) Centre, Institute of Psychiatry, Psychology and Neuroscience, Box P080, De Crespigny Park, SE5 8AF London, UK; b NIHR BioResource Centre Maudsley, NIHR Maudsley Biomedical Research Centre (BRC) at South London and Maudsley NHS Foundation Trust (SLaM) & Institute of Psychiatry, Psychology and Neuroscience (IoPPN), King’s College London; c Department of Psychosis Studies, Institute of Psychiatry, Kings College London, Box P092, De Crespigny Park, SE5 8AF London, UK; d Department of Psychological Medicine, Institute of Psychiatry, Kings College London, De Crespigny Park, SE5 8AF London, UK; e Department of Biostatistics and Health Informatics, Institute of Psychiatry, Psychology and Neuroscience, Box P080, De Crespigny Park, SE5 8AF London, UK; f Departments of Psychiatry and Medical Genetics, University of Alberta, 11361-87 Avenue, AB T6G 2E1, Edmonton, Canada

**Keywords:** First episode psychosis, Transcriptome, PANSS, Gene expression, Anti-psychotic medication, Immune system

## Abstract

**Background:**

Psychosis is a condition influenced by an interaction of environmental and genetic factors. Gene expression studies can capture these interactions; however, studies are usually performed in patients who are in remission. This study uses blood of first episode psychosis patients, in order to characterise deregulated pathways associated with psychosis symptom dimensions.

**Methods:**

Peripheral blood from 149 healthy controls and 131 first episode psychosis patients was profiled using Illumina HT-12 microarrays. A case/control differential expression analysis was performed, followed by correlation of gene expression with positive and negative syndrome scale (PANSS) scores. Enrichment analyses were performed on the associated gene lists. We test for pathway differences between first episode psychosis patients who qualify for a Schizophrenia diagnosis against those who do not.

**Results:**

A total of 978 genes were differentially expressed and enriched for pathways associated to immune function and the mitochondria. Using PANSS scores we found that positive symptom severity was correlated with immune function, while negative symptoms correlated with mitochondrial pathways.

**Conclusions:**

Our results identified gene expression changes correlated with symptom severity and showed that key pathways are modulated by positive and negative symptom dimensions.

## 1 Introduction

Psychosis is a debilitating disorder characterised by hallucinations, delusions and thought disorder, that affects approximately 4 in 1000 individuals in the UK each year (Kirkbride et al., 2012). It is a core feature of severe mental illnesses, such as schizophrenia and bipolar disorder, where treatment commonly involves the long-term use of anti-psychotic medication. However, the response to antipsychotics varies between patients, and is associated with the burden of significant side-effects. In order to find a tolerable treatment, it is often necessary for newly diagnosed patients to trial multiple antipsychotics. Developing a better understanding of psychosis subtypes could therefore benefit patients by identifying biomarkers that correlate with medication tolerance.

Schizophrenia and Bipolar disorder are both thought to be highly heritable, and recent genome wide association studies suggest that hundreds of common genetic variants contribute to disease risk (Ripke et al., 2014). The most prominent results from schizophrenia genetic studies have been associations to the major histocompatibility complex (MHC) locus, which implicates the immune system. Transcriptomic studies in blood have also consistently identified disruptions in immune pathways both in schizophrenia (Gardiner et al., 2013) and in bipolar disorder (Jansen et al., 2016). Recent meta analyses of transcriptome data have further strengthened these findings and found common and abnormal cytokine patterns in patients with chronic depression, bipolar disorder or schizophrenia (Goldsmith et al., 2016).

While psychosis is obviously closely linked to the brain, expression changes, are not necessarily linked to brain tissue. Peripheral gene expression changes can plausibly be detected in blood, especially in the case of the immune system. In a meta-analysis of Schizophrenia transcriptomics (Hess et al., 2016) that pooled 18 previously published blood and brain-based studies the authors found significant overlap in dysregulated pathways between both tissues, including the upregulation of innate immune pathways. Blood based transcriptomic studies have the additional advantage of capturing complex gene environment interactions, at key points during disease progression. This includes first episode psychosis which is of special interest since patients will get treatment for the first time, following an acute episode of symptoms.

However, there are few studies that focus specifically on the transcriptome of first episode psychosis (FEP) patients, when they first encounter mental health services. One notable exception is a Singaporean study which identified a 400 gene signature to distinguish FEP patients and healthy controls (Lee et al., 2012), however this study was limited to 26 patients and did not distinguish between positive and negative symptom dimensions.

To investigate the potential relationship between psychosis specific gene expression changes, and diagnostic/symptom dimensions, we make use of the Genetics and Psychosis (GAP) study (Di Forti et al., 2009). GAP contains transcriptome data derived from the whole blood of healthy controls (HC), as well as FEP patients. While these patients did receive anti-psychotic medication where necessary, they were recruited within one week of admission to hospital, and exposure to medication was therefore limited compared to chronic psychosis sufferers' who may have taken medication continuously for many years.

We report the results of a case-control differential gene expression (DGE) and pathways analysis using the GAP transcriptome data. This is followed by investigating the correlation between psychosis pathways and symptom dimensions. Finally, we compare how the disruption of pathways differs in FEP patients who qualify for a Schizophrenia diagnosis and those who do not.

## 2 Methods and materials

### 2.1 Ethics

The Genetics and Psychotic Disorders (GAP) Study received ethical approval from the South London and Maudsley NHS Foundation Trust (SLaM), as well as from the Institute of Psychiatry, Psychology and Neuroscience Local Research Ethics Committee, IOPPN/SLaM research ethics approval number: 135/05. Informed written consent was obtained from all participants in the study by the GAP team.

### 2.2 Study design and participants

As part of the GAP study (Di Forti et al., 2009, Di Forti et al., 2015), Patients aged 18 to 65 years who presented with first-episode psychosis at the inpatient units of SLaM were approached for recruitment. Patients were invited to participate if they met the ICD-10 criteria for a diagnosis of non-affective (F20 to F29) or affective (F30 to F33) psychosis (Guest et al., 2014), validated by administration of the Schedules for Clinical Assessment in Neuropsychiatry (SCAN) (Aboraya et al., 1989), they were re-contacted after the start of treatment.

Between May 1, 2005, and May 31, 2011, 461 patients with first-episode psychosis were recruited. The cohort consisted of a diverse multi-ethnic population. Further patient information, blood samples and genetic ancestry were acquired as described previously (Di Forti et al., 2012). During the same period, a total of 389 control individuals were recruited. These individuals were aged 18–65 years and were similar to the local population regarding gender, ethnic origin, education, employment status and socio-economic status. Recruitment of controls was done using Internet and newspaper advertisements and by distributing leaflets at train stations, shops, and job centres. Volunteers were administered the Psychosis Screening Questionnaire (Bebbington and Nayani, 1996) and were excluded if they met the criteria for a psychotic disorder or if they reported a previous diagnosis of psychotic illness.

The OPCRIT system (Rucker et al., 2011) was used to assign ICD-10 and DSM-IV diagnostic categories in a systematic way. For subset analyses including diagnosis, patients who fit the diagnostic criteria for schizophrenia under either the ICD or DSM system were assigned to the schizophrenia (Scz) group, while all other patients were assigned to the other psychosis group (OP).

### 2.3 RNA processing and quality control

Whole blood samples were collected using PAXgene tubes for RNA, from a subset of GAP participants (227 cases and 168 controls) within the first two weeks of recruitment. Samples were run at the NIHR Maudsley Biomedical Research Centre for Mental Health (BRC-MH) microarray facility at the SGDP, Institute of Psychiatry, Psychology and Neuroscience, King's College London, UK. Microarrays where run in accordance with the manufacturer's protocol using Illumina HT-12 V4 bead-chips (Illumina, USA).

All analysis was performed using R version 3.1.2. Pre-processing of the data was performed using an in-house developed pipeline.

The pipeline takes raw gene expression data exported from Illumina's Genomestudio, performs background correction (Xie et al., 2009) using negative bead expression levels in order to correct for noise. Lumi (version 2.22.1 Du et al. (2008)) was used to log base 2 transform the data followed by robust spline normalization (Du et al., 2008). Outlying samples were iteratively identified using fundamental network concepts and removed, following the methods described by Oldham et al. (2012).

In order to reduce the influence of batch effects we adjusted for technical categorical variables using multiple linear regression and ComBat (Leek et al., 2010). Other Continuous technical artefacts were accounted for by taking the first principal component across housekeeping and undetected probes and regressing this against technical variables. Four variables were significantly associated with PC1, these were as follows: “The date that Samples were thawed”, “Concentration of initial RNA”, “Concentration of labelled cRNA” and “Date of RNA purification”. They were regressed against expression for each probe, and the mean adjusted residuals where taken forward for all further analysis. The RNA integrity number (RIN) was include in the list of variables but was not significantly correlated with the first principle component. We did not use a RIN cut-off, to preserve sample size, as a result 24 samples had a RIN between 8 and 5.

The expression data was further tested for hidden covariates using surrogate variable analysis (Leek and Storey, 2007). FEP and Control status was included in the model to preserve the biological signal between groups. Following this we compared recorded gender with gender determined by XIST and PRKY probes and excluded samples that showed a mismatch. Finally, we excluded all probes that could not be reliably detected in 80% of the samples in at least one diagnostic group. We used the R package CellMix version 1.6 (Gaujoux and Seoighe, 2013), to test for potential significant differences in whole blood cell populations between cases and controls and these were included in limma models.

### 2.4 Differential expression analysis

Standard Differential expression analysis was performed using the R package LIMMA (Smyth, 2004) (version 3.26.8). Prior to analysis we accounted for potential covariates by performing multiple linear regression on all probes with CellMix proportions, age, sex and ethnicity as independent variables. Mean adjusted residuals were used in all subsequent analysis. Probes were declared significantly differentially expressed if the FDR adjusted q-value was <0.05 and the absolute log fold change was above 0.1. Differential expression was firstly tested in first episode psychosis (FEP) group vs healthy controls followed by a subgroup analysis comparing the Schizophrenia (Scz) and Other Psychosis (OP) groups with healthy controls.

### 2.5 Symptom severity

Illness severity was assessed by using the Positive and Negative Syndrome Scale (PANSS) (Kay et al., 1987), which consists of 30 questions, each ranging from 1 (not present) to 7 (extreme). The PANSS is subdivided into 3 subscales with the positive and negative subscales containing 7 questions each and the general psychopathology subscale containing 16 questions.

Correlation between patient PANSS scores and differentially expressed probes was calculated using Pearson's r, implemented by the rcorr function in the Hmisc r package. Probe correlations with a p-value below 0.05 were filtered out. Remaining probes were used in gene enrichment analysis.

### 2.6 Gene enrichment analysis

All enrichment analysis was performed using the UserListEnrichment function of the WGCNA package (Langfelder and Horvath, 2008). Enrichment analysis, for the results of the LIMMA analysis, was performed by testing differentially expressed probes (q-value≤0.05 and logFC >0.1). All probes that did not pass the q-value threshold were included as background.

In all cases KEGG (Kanehisa et al., 2004) and GO (Ashburner et al., 2000) databases taken from the Enrichr website were used (http://amp.pharm.mssm.edu/Enrichr/st accessed 05. July.2016). The databases used were KEGG 2016, GO Molecular Function 2015, GO Cellular Component and GO Biological Process 2015. Additionally, internal lists from the UserListEnrichment function in WGCNA were used. These were Brain Modules (useBrainLists), Brain Region Markers (useBrainRegionMarkers) and Blood Atlases (useBloodAtlases). Gene lists specifically compiled for Psychosis were included from publications by Purcell et al. (2014) and Pirooznia et al. (2016). Result categories that contained <20 overlapping probes were filtered out.

### 2.7 Anti-psychotic medication

Psychosis patients were received standard care (including anti-psychotics where necessary) until they were stabilised, and blood samples were then obtained within the first two weeks of recruitment. To assess the effect of medication on gene expression, we first identified the antipsychotics medication that patients were taking at the time of blood collection. We grouped FEP samples based on the three most common antipsychotic categories which were as follows: Olanzapine (N = 46), Risperidone (N = 27) and antipsychotic free (N = 18). We performed differential expression analysis between the three medication groups and controls.

## 3 Results

### 3.1 Sample characteristics

Demographics data is shown in Table 1. Transcriptomic data was available for 280 samples, 149 of these corresponded to healthy controls (HC), and 131 to first episode psychosis (FEP) patients. The FEP group further consisted of 68 patients with a Schizophrenia diagnosis and 63 patients with other psychoses, according to the OPCRIT system (Rucker et al., 2011). The other psychosis group primarily fit the DSM-IV categories of mania (n = 16), schizo-affective disorder (n = 15), depression with psychosis (n = 12) and psychosis NOS (n = 10) (see Supplementary Table 1). FEP with a diagnosis of Schizophrenia was on average younger than other FEP patients and controls. FEP patients had similar exposures to antipsychotic medication, independently from diagnosis, with Olanzapine and Risperidone being the most common. FEP on average had mild to moderate symptoms according to PANSS assessment (Leucht et al., 2005), with those with a diagnosis of schizophrenia presenting with slightly higher scores for positive and negative symptoms subscales than FEP with other psychoses (see Table 1).

### 3.2 Differential expression analysis

We identified 978 differentially expressed genes between FEP and HC, 509 of these were upregulated and 469 were downregulated in FEP. The most significantly upregulated genes were SUMO3, CAMP, DEFA1B, DEFA1 and DEFA3. The most significantly downregulated genes were HNRNPUL2, RBM14, TMEN69, SCAP and FAM110A (see Table 2, and Supplementary Table 2).

Gene Enrichment analysis identified 116 significant pathways. They overlapped substantially, and fundamentally corresponded to just a handful of distinct gene lists and pathways, including ribosomal function/transcription regulation, immune signalling, and energy metabolism (see Table 3, Supplementary Table 3).

All 978 differentially expressed probes were correlated with the PANSS subscales. For the positive and negative subscales, we identified 120 and 37 significantly correlated probes respectively (Supplementary Table 4). Gene enrichment analysis of probes found positive symptoms were correlated with immune pathways (NF-κB, Ras and cytokine signalling), while negative symptoms were correlated to ribosomal pathways (see Table 4 and Supplementary Table 4).

### 3.3 Differences between schizophrenia and other psychosis

In order to explore differences in FEP subgroups we replicated analyses by independently comparing the Schizophrenia (Scz) and Other Psychosis (OP) groups with healthy controls (HC). Analysis using a Scz vs OP design was also performed, but no significantly differentially expressed genes were identified (results not shown).

**Table 1:** Demographics.

	Healthy control (n = 149)	First Episode Psychosis (n = 131)		
		Schizophrenia (n = 68)	Other psychosis (n = 63)	p-value
Sex = MALE (%)	86 (57.7)	45 (66.2)	31 (49.2)	0.145
Age (mean (sd))	29.87 (10.53)	26.59 (7.67)	30.03 (9.22)	0.047

Ethnicity (%)				0.102
Asian	10 (6.7)	6 (8.8)	6 (9.5)	
Black	43 (28.9)	33 (48.5)	22 (34.9)	
Other	10 (6.7)	5 (7.4)	5 (7.9)	
White	86 (57.7)	24 (35.3)	30 (47.6)	

PANSS scores (mean (sd))				
Positive scale		16.70 (6.88)	15.49 (6.00)	0.344
Negative scale		17.20 (6.90)	14.76 (6.08)	0.065

Anti-psychotic type (%)				0.6
Antipsychotic free		9 (13.2)	9 (14.3)	
Olanzapine		22 (32.4)	24 (38.1)	
Risperidone		14 (20.6)	13 (20.6)	
Amisulpride		0 (0.0)	1 (1.6)	
Aripiprazole		9 (13.2)	3 (4.8)	
Haloperidol		2 (2.9)	4 (6.3)	
Quetiapine		4 (5.9)	2 (3.2)	
Sulpiride		1 (1.5)	0 (0.0)	
Trifluoperazine		1 (1.5)	0 (0.0)	
Unknown		6 (8.8)	7 (11.1)	

Table of Demographics for healthy control (HC) and first episode psychosis (FEP) groups. FEP patients are separated by diagnosis into Schizophrenia and Other Psychosis. The study included 280 individuals. Schizophrenia patients were found to be slightly younger than controls and other patients. No significant difference was found for Gender, Ethnicity, PANSS scores or Anti-psychotic type. Pvalues were calculated using the chi-square test for categorical variables, and t-test for continues variables.

**Table 2:** Top differentially expressed probes.

Gene	FEP vs HC	Scz vs HC	OP vs HC	Chr
Top 50 Up-regulated - logFC (q-value)				
CAMP	0.66 (<0.001)	0.94 (<0.001)	0.38 (0.058)	3
DEFA1B	0.97 (<0.001)	1.25 (<0.001)	0.69 (0.042)	8
DEFA3	0.95 (<0.001)	1.24 (<0.001)	0.66 (0.046)	8
DEFA1	0.87 (<0.001)	1.1 (<0.001)	0.64 (0.041)	8
C9ORF72	0.24 (<0.001)	0.3 (<0.001)	0.19 (0.041)	9
CLNS1A	0.18 (<0.001)	0.23 (<0.001)	0.13 (0.049)	11
SUMO3	0.11 (<0.001)	0.12 (<0.001)	0.1 (0.022)	21
TMEM170B	0.3 (<0.001)	0.36 (<0.001)	0.25 (0.037)	6
PSMC2	0.14 (<0.001)	0.18 (<0.001)	0.1 (0.050)	7
HBXIP	0.1 (<0.001)	0.13 (<0.001)	0.07 (0.053)	1
PARL	0.1 (<0.001)	0.12 (<0.001)	0.07 (0.048)	3
IFNGR1	0.16 (<0.001)	0.19 (<0.001)	0.12 (0.050)	6
GLRX	0.24 (<0.001)	0.29 (<0.001)	0.19 (0.042)	5
IDH1	0.14 (<0.001)	0.16 (<0.001)	0.12 (0.036)	2
TCEB1	0.14 (<0.001)	0.16 (<0.001)	0.11 (0.041)	8
CCPG1	0.17 (<0.001)	0.21 (<0.001)	0.13 (0.046)	15
LYPLAL1	0.18 (<0.001)	0.2 (<0.001)	0.16 (0.037)	1
SLC30A9	0.18 (<0.001)	0.2 (<0.001)	0.15 (0.037)	4
TMBIM4	0.08 (<0.001)	0.1 (<0.001)	0.07 (0.044)	12
FAM96A	0.25 (<0.001)	0.29 (<0.001)	0.2 (0.040)	15
FAM45A	0.13 (<0.001)	0.16 (<0.001)	0.1 (0.048)	10
GNG10	0.31 (<0.001)	0.39 (<0.001)	0.24 (0.053)	9
C14ORF100	0.15 (<0.001)	0.17 (<0.001)	0.12 (0.040)	14
COX7A2	0.22 (<0.001)	0.27 (<0.001)	0.18 (0.046)	6
TAF7	0.2 (<0.001)	0.24 (<0.001)	0.17 (0.040)	5
MAP2K1IP1	0.21 (<0.001)	0.25 (<0.001)	0.17 (0.046)	4
CRLS1	0.21 (<0.001)	0.24 (<0.001)	0.17 (0.041)	20
COX7A2L	0.19 (<0.001)	0.21 (<0.001)	0.16 (0.037)	2
ATP5C1	0.24 (<0.001)	0.29 (<0.001)	0.2 (0.045)	10
FBXL5	0.13 (<0.001)	0.15 (<0.001)	0.1 (0.049)	4
UQCRQ	0.28 (<0.001)	0.33 (<0.001)	0.23 (0.046)	5
BNIP2	0.18 (<0.001)	0.19 (<0.001)	0.16 (0.037)	15
SLC35A1	0.19 (<0.001)	0.23 (<0.001)	0.15 (0.059)	6
RPSA	0.16 (<0.001)	0.2 (<0.001)	0.12 (0.061)	3
LYST	0.14 (<0.001)	0.16 (<0.001)	0.12 (0.046)	1
WDR61	0.13 (<0.001)	0.16 (<0.001)	0.11 (0.044)	15
SRP9	0.2 (<0.001)	0.22 (<0.001)	0.18 (0.038)	1
VBP1	0.22 (<0.001)	0.25 (<0.001)	0.19 (0.041)	X
PCMT1	0.14 (<0.001)	0.14 (<0.001)	0.13 (0.036)	6
TMX1	0.25 (<0.001)	0.29 (<0.001)	0.2 (0.048)	14
SLC44A1	0.18 (<0.001)	0.19 (<0.001)	0.16 (0.036)	9
PRDX3	0.13 (<0.001)	0.14 (<0.001)	0.12 (0.037)	10
COX17	0.13 (<0.001)	0.15 (<0.001)	0.11 (0.042)	3
KIAA1600	0.19 (<0.001)	0.22 (<0.001)	0.15 (0.050)	10
PIGY	0.17 (<0.001)	0.2 (<0.001)	0.15 (0.044)	4
COMMD3	0.17 (<0.001)	0.21 (<0.001)	0.14 (0.049)	10
KBTBD11	0.16 (<0.001)	0.19 (<0.001)	0.13 (0.050)	8
TMEM14B	0.16 (<0.001)	0.19 (<0.001)	0.13 (0.055)	6
VAMP7	0.15 (<0.001)	0.16 (<0.001)	0.14 (0.036)	XY
LDHA	0.11 (<0.001)	0.11 (<0.001)	0.11 (0.036)	11

Top 50 Down-regulated - logFC (q-value)				
RBM14	−0.13 (<0.001)	−0.17 (<0.001)	−0.09 (0.046)	11
HNRNPUL2	−0.19 (<0.001)	−0.23 (<0.001)	−0.14 (0.040)	11
GABPB2	−0.09 (<0.001)	−0.11 (<0.001)	−0.06 (0.053)	1
FAM110A	−0.16 (<0.001)	−0.19 (<0.001)	−0.12 (0.046)	20
SCAP	−0.14 (<0.001)	−0.17 (<0.001)	−0.11 (0.046)	3
PNPT1	−0.11 (<0.001)	−0.14 (<0.001)	−0.08 (0.050)	2
RASGRP2	−0.12 (<0.001)	−0.15 (<0.001)	−0.09 (0.050)	11
ZC3H5	−0.11 (<0.001)	−0.13 (<0.001)	−0.08 (0.057)	
TMEM69	−0.11 (<0.001)	−0.13 (<0.001)	−0.1 (0.037)	1
CLSTN1	−0.16 (<0.001)	−0.2 (<0.001)	−0.12 (0.058)	1
GANAB	−0.12 (<0.001)	−0.15 (<0.001)	−0.09 (0.054)	11
DENND4B	−0.12 (<0.001)	−0.14 (<0.001)	−0.09 (0.046)	1
ZNF296	−0.16 (<0.001)	−0.19 (<0.001)	−0.13 (0.042)	19
KIAA1267	−0.1 (<0.001)	−0.13 (<0.001)	−0.08 (0.051)	17
CXXC1	−0.12 (<0.001)	−0.14 (<0.001)	−0.1 (0.046)	18
POM121C	−0.13 (<0.001)	−0.16 (<0.001)	−0.1 (0.049)	7
RANGAP1	−0.12 (<0.001)	−0.15 (<0.001)	−0.1 (0.050)	22
STIP1	−0.14 (<0.001)	−0.17 (<0.001)	−0.1 (0.060)	11
ITPKB	−0.13 (<0.001)	−0.16 (<0.001)	−0.1 (0.055)	1
CD97	−0.16 (<0.001)	−0.2 (<0.001)	−0.13 (0.053)	19
HGS	−0.1 (<0.001)	−0.13 (<0.001)	−0.08 (0.060)	17
SUPT5H	−0.14 (<0.001)	−0.16 (<0.001)	−0.11 (0.042)	19
UBQLN4	−0.12 (<0.001)	−0.14 (<0.001)	−0.1 (0.044)	1
SPG7	−0.11 (<0.001)	−0.13 (<0.001)	−0.08 (0.058)	16
RAB11FIP1	−0.14 (<0.001)	−0.16 (<0.001)	−0.12 (0.046)	8
TRIM28	−0.14 (<0.001)	−0.16 (<0.001)	−0.11 (0.053)	19
WDR23	−0.12 (<0.001)	−0.13 (<0.001)	−0.1 (0.041)	14
GPS1	−0.09 (<0.001)	−0.1 (<0.001)	−0.08 (0.046)	17
FBXO46	−0.16 (<0.001)	−0.18 (<0.001)	−0.15 (0.041)	19
TLN1	−0.21 (<0.001)	−0.24 (<0.001)	−0.18 (0.044)	9
ABCF1	−0.11 (<0.001)	−0.13 (0.001)	−0.09 (0.060)	6
ORC6L	−0.1 (<0.001)	−0.11 (0.001)	−0.08 (0.046)	16
UBA1	−0.17 (<0.001)	−0.2 (0.001)	−0.13 (0.061)	X
SRRM2	−0.11 (<0.001)	−0.14 (0.001)	−0.09 (0.057)	16
PDPR	−0.21 (<0.001)	−0.24 (0.001)	−0.18 (0.047)	
XRCC6	−0.13 (<0.001)	−0.14 (0.001)	−0.11 (0.046)	22
LBA1	−0.18 (<0.001)	−0.21 (0.001)	−0.16 (0.048)	3
GCN1L1	−0.12 (<0.001)	−0.14 (0.001)	−0.1 (0.052)	12
C21ORF58	−0.12 (<0.001)	−0.14 (0.002)	−0.1 (0.061)	21
EMD	−0.09 (<0.001)	−0.1 (0.002)	−0.07 (0.059)	X
ST3GAL1	−0.14 (<0.001)	−0.15 (0.002)	−0.13 (0.037)	8
TSSC4	−0.11 (<0.001)	−0.11 (0.002)	−0.1 (0.037)	11
EDC4	−0.1 (<0.001)	−0.11 (0.002)	−0.1 (0.037)	16
CORO7	−0.12 (<0.001)	−0.14 (0.002)	−0.11 (0.048)	16
WASF2	−0.18 (<0.001)	−0.2 (0.002)	−0.17 (0.040)	1
UBA52	−0.11 (<0.001)	−0.12 (0.002)	−0.1 (0.044)	19
NRGN	−0.3 (<0.001)	−0.33 (0.002)	−0.26 (0.048)	11
AP1G2	−0.11 (<0.001)	−0.11 (0.002)	−0.1 (0.041)	14
KPNA6	−0.12 (0.001)	−0.14 (0.002)	−0.1 (0.057)	1
PBX2	−0.12 (<0.001)	−0.13 (0.002)	−0.11 (0.041)	6

Table of logFC and q-value of top up and down regulated probes for 3 comparisons (FEP vs HC, SCZ vs HC and OP vs HC) following differential expression analysis. Each comparison contains the logFC value followed by the q-value in brackets. Genes were included if the FDR adjusted q-value was less than 0.05 and the absolute log fold change was above 0.1 in all 3 comparisons. For a complete list of probes see supplementary table 2.

Table of logFC and q-value of top up and down regulated probes for 3 comparisons (FEP vs HC, SCZ vs HC and OP vs HC) following differential expression analysis. Each comparison contains the logFC value followed by the q-value in brackets. Genes were included if the FDR adjusted q-value was less than 0.05 and the absolute log fold change was above 0.1 in all 3 comparisons. For a complete list of probes see supplementary table 2.

**Table 3:** Enriched Gene Sets: FEP vs HC.

Gene set	Library	P-value	#	Genes
*Kyoto Encyclopedia of Genes and Genomes*				
Ribosome	KEGG	<0.001	46	FAU, MRPL11, MRPL3, RPL17, RPL21, UBA52
Oxidative phosphorylation	KEGG	<0.001	28	ATP5C1, COX17, NDUFA1, PPA1, SDHD, UQCRH
Parkinson's disease	KEGG	<0.001	27	ATP5C1, COX17, NDUFA1, PPA1, SDHD, UQCRH
Alzheimer's disease	KEGG	<0.001	26	ATP5C1, COX17, NDUFA1, PPA1, SDHD, UQCRH
Huntington's disease	KEGG	0.001	26	ATP5C1, COX17, NDUFA1, PPA1, SDHD, UQCRH
*Gene Ontology: Biological Process*				
Cytokine production	GOBP	0.007	14	CASP1, CD46, G6PD, IL1B, PIK3CD, TBK1, TNFAIP8
T cell receptor	GOBP	0.009	16	CARD11, CD3D, FYN, LAT, NCK1, RBCK1, UBE2N
Leukocyte activation	GOBP	0.02	37	AIF1, ANXA1, CD3E, CD48, CD7, CD79A, CD93, CXCR4, FYN, FZD7, GAPT, ITGAL, LAT, LYL1, SP3, VAMP7
Regulation of NF-kB	GOBP	0.041	25	BIRC2, CARD11, CASP1, CD36, CXXC5, FYN, HSPB1, IL1B, IRF3, RBCK1, UBE2N
Defense response to Bacteria	GOBP	0.043	11	CAMP, CD36, DEFA1, FAU, HIST2H2BE, MYO1F, PGLYRP1, RPL39, TBK1, TNFAIP8

*Gene Ontology: Cellular Component*				
Cytosol	GOCC	<0.001	238	ATG3, BCR, DCP2, FYN, GMIP, GNAS, IL1B, IRF3, LYST, NDE1, NME1, PARK7, TBK1, UBA1, USP7
Ribosomal subunit	GOCC	<0.001	45	FAU, FXR2, MRPL3, RPL11, RPS15A, RPS3, RPS9
Nucleolus	GOCC	<0.001	127	AATF, CD79B, HIF1A, ILF3, IP6K1, JUND, NIN, PCNA, PTEN, RNF7, RPL11, STAT3, ZFR
Mitochondrion	GOCC	0.002	90	ATP5L, COQ5, COX7C, CYBB, ILF3, NME1, NUBPL, OAS2, PARK7, REXO2, TPP1, UCP2
Lysosome	GOCC	0.01	29	CTNS, CTSA, CTSB, CTSZ, CXCR4, FUCA1, GALC, HEXB, LAT, SRGN, TPP1, USP4

*Gene Ontology: Biological Process*				
Ribosome	GOMF	<0.001	45	FAU, MRPL11, RPL27, RPS3, RPS3A, RPSA
Ion transport	GOMF	<0.001	18	ATP5C1, ATP5E, COX6A1, NDUFA4, SLC9A1, UQCRH

*Blood Module Library (WGCNA)*				
Red Blood Cell	Blood	<0.001	170	ADD3, BAT3, CD48, IL8RB, JUND, LAMP2, LDHA, LYST, MED1, PARK7, STAT3, TBK1, UBN1
Blood Platelets	Blood	<0.001	39	ACTN1, ANXA1, BCR, GNAS, GP9, JUND, LCN2, NRGN, TAGLN2, VCL

*Brain Module Library (WGCNA)*				
Post Synaptic Density Proteins	Brain	<0.001	133	AGL, BAT3, BCR, FASN, FYN, G6PD, HGS, MSN, MTDH, MYH9, NME1, SEPT5, SND1, TAGLN2, TBK1, TLN1, TUBB2C, UBA1, VAMP2
Pyramidal Neurons (Amygdala)	Brain	<0.001	37	ACSL4, CRBN, DDR1, ENY2, GMFB, HDAC2, MATR3, NME1, PCNA, TBK1, UBE3A
Down in Alzheimer's	Brain	<0.001	80	ACP1, ATP5O, ELMO1, GLO1, GMFB, GNAS, HEXB, ICA1, NME1, PARK7, STX7, TAF7, UBL3
Glutamatergic synapse	Brain	<0.001	49	ARF3, ATG3, BAT3, CABIN1, CENTB2, CPD, CTSD, DEK, GCA, GPR137, HGS, NRGN, PRNP, RARA, SPOCK2, SPTAN1, SRP9, TBCA, TIA1, UBE1, VAMP2
CD40 stimulated Microglial cells	Brain	<0.001	58	ACP1, BCR, CARM1, DDR1, FYN, ILF3, IRF3, NME1, SLC9A1, TRIM28, UBE2L3, VAMP2

Table of select gene sets enriched with differentially expressed (n = 978) genes between FEP and HC. P-values are Bonferroni corrected. Total number of DE genes present in each gene set are indicated in the # column. List of gene sets and gene symbols is not exhaustive, for complete results see supplementary table 2.

Table of select gene sets enriched with differentially expressed (n = 978) genes between FEP and HC. P-values are Bonferroni corrected. Total number of DE genes present in each gene set are indicated in the # column. List of gene sets and gene symbols is not exhaustive, for complete results see supplementary table 2.

**Table 4:** Gene sets correlated with symptom severity.

Gene set	Library	P-value	#	Genes
*PANSS Positive symptom correlated pathways*				
Measles	KEGG	0.0034	8	CD3E, CD46, FYN, IFNGR1, IL1B, IL2RB, IRF3, TBK1
Cytokine production	GOBP	0.009	6	CASP1, CD46, EOMES, IL1B, PTGS2, TBK1
Regulation of NF-kB signalling	GOBP	0.0168	8	CASP1, CXXC5, FYN, IL1B, IRF3, PELI1, PTGS2, TBK1
Red Blood Cell	Blood	0.0199	18	BAZ2B, CD46, CKLF, COX7A2, DYNLT1, GALC, GNG10, IFNGR1, LYST, PELI1, PTGS2, RAB5A, RNF13, SHOC2, SRGN, TBK1, TNFAIP6, TXN
Ras signalling	KEGG	0.0219	9	CSF1R, GNG10, LAT, RAB5A, RASAL3, RASGRP2, SHOC2, TBK1, ZAP70
Cytosol	GOCC	0.035	31	ALDOC, CAPZA2, CASP1, DPYD, FASN, FYN, IDH1, IL1B, IRF3, ITPKB, LYST, MATK, OSBPL7, PDE7A, PELI1, PTEN, RAP2C, TBK1, TXN, UBE2E1, USP7

*PANSS Negative symptom correlated pathways*				
Viral transcription	GOBP	<0.001	8	RPL11, RPL21, RPL35, RPL4, RPL5, RPS3, RPS5, RPS6
Cytosolic part	GOCC	<0.001	9	AHR, RPL11, RPL21, RPL35, RPL4, RPL5, RPS3, RPS5, RPS6
Ribosome	GOMF	<0.001	8	RPL11, RPL21, RPL35, RPL4, RPL5, RPS3, RPS5, RPS6
Ribosome	KEGG	<0.001	8	RPL11, RPL21, RPL35, RPL4, RPL5, RPS3, RPS5, RPS6
Mitochondria	Brain	0.0071	6	COX6C, NDUFS5, PPA2, RPL11, RPL4, RPL5

Table of gene sets enriched with DE genes significantly correlated to positive or negative symptoms in FEP. The 120 and 37 DE genes significantly correlated, respectively, with Positive and Negative symptoms were used for gene enrichment analysis. Significant pathways are shown separately for the two symptom dimensions. P-values are Bonferroni corrected. Total number of DE genes overlapping with gene sets are indicated in the # column. For complete results, including genes correlated with symptom severity, see supplementary table 4.

Table of gene sets enriched with DE genes significantly correlated to positive or negative symptoms in FEP. The 120 and 37 DE genes significantly correlated, respectively, with Positive and Negative symptoms were used for gene enrichment analysis. Significant pathways are shown separately for the two symptom dimensions. P-values are Bonferroni corrected. Total number of DE genes overlapping with gene sets are indicated in the # column. For complete results, including genes correlated with symptom severity, see supplementary table 4.

**Fig. 1:**
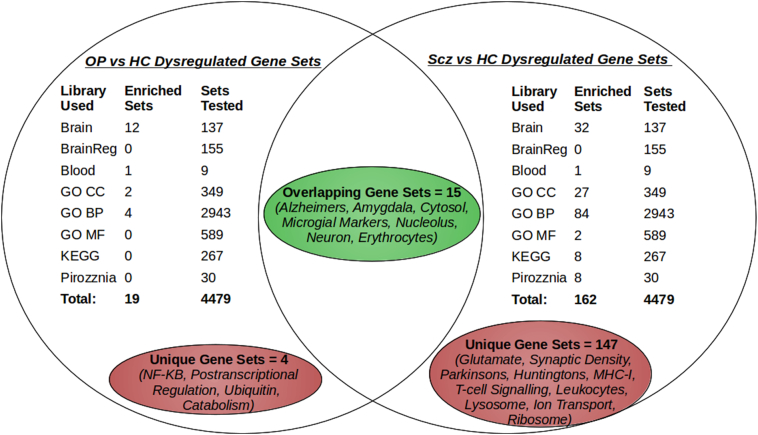
Summary of Enrichment results for Schizophrenia and Other Psychosis. Venn diagram summarising and comparing Gene Set Enrichment results between Schizophrenia (Scz) and Other Psychoses (OP) groups. Differentially expressed genes from comparisons with healthy controls (HC) were used. A total of 8 gene set libraries with a combined 4479 categories were used. The Bonferroni corrected p-value cut-off for gene sets was 0.05. Pathways and functions associated with significant gene sets are highlighted in red (for pathways unique to a group) and green (for pathways present in both groups). The FEP vs HC comparison is not included since the results are a combination of the Scz and OP results.

**Fig. 2:**
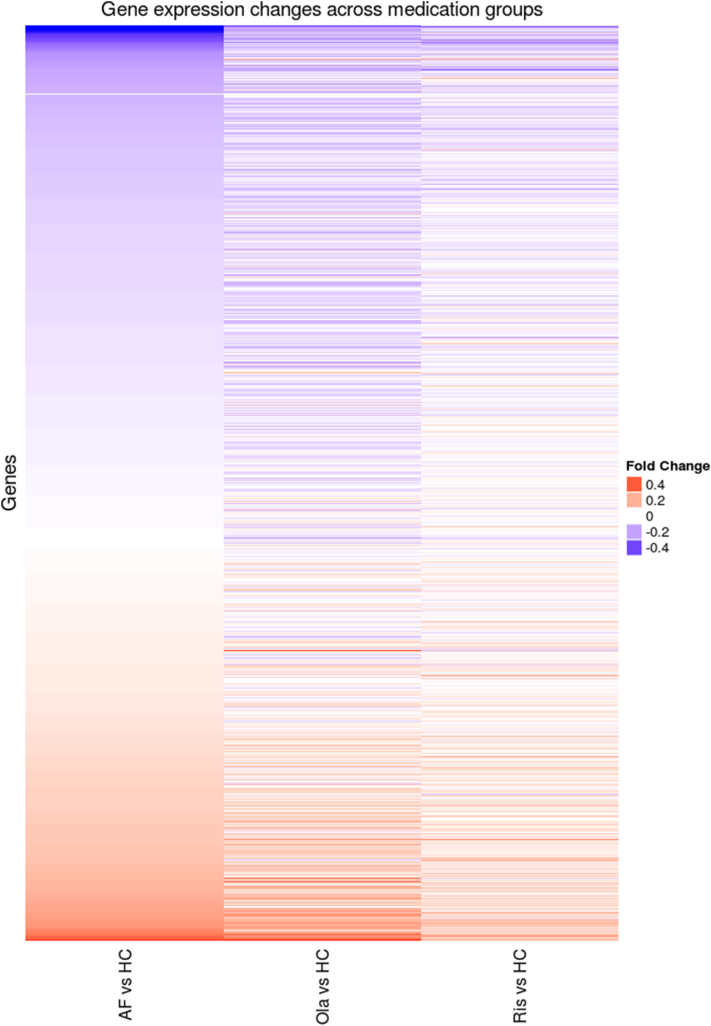
Gene Expression Change across Medication Groups. Heatmap comparing differential expression, between anti-psychotic medication groups and healthy controls. Anti-psychotic free (AF, N = 18), Olanzapine (Ola, N = 46) and Risperidone (Ris, N = 27) patients were compared to healthy controls (HC, N = 149). Results were subset to significant DE genes from the full FEP vs HC comparison, and fold change was plotted. The direction of gene expression tends to be preserved in the three comparisons.

Differential expression analysis of Scz vs HC identified 1276 differentially expressed genes, while the OP vs HC differential expression resulted in 284 significant genes (see Supplementary Table 2). Gene Enrichment identified 162 pathways in the Scz group, and 19 in the OP group, 15 of which were present in both groups. The 4 pathways unique to the OP group were related to NF-κB signalling, catabolic processes and transcriptional regulation of gene expression (see Fig. 1, Supplementary Table 3).

Differentially expressed genes were again correlated with PANSS subscales, and pathway analysis was performed. In the Scz group we identified 202 and 37 probes that were significantly correlated with positive and negative symptoms, respectively, while in the OP group we found 5 and 9 probes respectively (Supplementary Table 4).

Pathway analysis of correlated probes was not performed for the OP group due to the low number of probes. For the Scz group, pathway analysis identified 11 pathways correlated with positive symptoms including immune pathways (lymphocyte/leukocyte activation, cytokine production), the cytosol, response to protein misfolding, exosome transport and four brain related pathways (neuronal cytoplasm, synaptome, glutamatergic synapse and post synaptic density). For negative symptoms the only pathway identified was related to RNA catabolism (Supplementary Table 4).

### 3.4 Anti-psychotic medication

In the differential expression comparison between the three medication groups (Olanzapine, Risperidone, antipsychotic free [AF]) and controls, we identified no significant DE genes in the Risperidone - HC comparison, but did find 1132 and 338 DE genes in the Olanzapine - HC and AF - HC comparisons respectively (see Supplementary Table 5). To visualise the effect of medication, we used the 978 significant DE genes from the full FEP – HC comparison, and plotted the fold change for these genes in the three medication subsets (see Fig. 2), The direction (meaning up or down regulation) of differential expression compared to healthy controls was generally preserved across the three groups, but the magnitude (or fold change) of probes tended to be greater for the antipsychotic free group than for the olanzapine or risperidone groups, as can be seen in Fig. 2. In addition, no differentially expressed genes were found when comparing either of the antipsychotic groups with the antipsychotic free group.

## 4 Discussion

### 4.1 First episode psychosis patients show deregulation in immune pathways

Our results suggest an immune deregulation component in psychosis patients, which is in line with expectations due to previous GAP studies (Mondelli et al., 2011, Mondelli et al., 2015; Di Nicola et al., 2013). Notably we find an upregulation of the antimicrobial α-defensins (DEFA1B, DEFA1 and DEFA3), which was independently reported for schizophrenia in a transcriptome study by Gardiner et al. (2013). Gene enrichment analysis revealed dysregulation in multiple pathways that are related to oxidative stress, NF-κB signalling and the Ribosome, which is consistent with the results by Hess et al. (2016).

### 4.2 Patients meeting criteria for a diagnosis of schizophrenia have a more pronounced blood signature

We identified 1276 differentially expressed genes in the Scz vs HC comparison. This number was higher than what was found in similar gene expression studies of chronic schizophrenia (Gardiner et al., 2013), and may be explained by blood samples, in this study, being taken soon after patients were admitted to hospital, since the samples used in this study are first episode. As such the reported gene expression signature may more closely reflect the expression changes during acute psychosis, rather than during remission, and they might be less confounded by the effects of long-term antipsychotic treatment.

Comparing the result from Scz and OP analyses, we find more than fourfold as many differentially expressed genes, and pathways in the Schizophrenia group, probably reflecting the higher homogeneity. Interestingly 215 of the 284 dysregulated genes in the OP group, were also found in the Schizophrenia group. Furthermore, the direction of change was the same in both groups, although the log fold change in expression was lower for OP (see Table 2). This suggests some common psychosis gene expression signature across diagnostic categories.

Interestingly 12 of the 19 significant pathways in the OP group are from brain specific pathway libraries, and all 12 were also identified in the Scz group (Fig. 1, Supplementary Table 3). A recent study pooling 18 schizophrenia transcriptome cohorts found that 50% of blood enriched pathways were also enriched in brain tissue (Hess et al., 2016). While we do not suggest that these results accurately reflect gene expression patterns in the brains of patients, it is interesting to note that the overlapping brain pathways include Alzheimer's Disease, the Amygdala and Microglial markers. This might be consistent with a neuro-inflammatory model of psychosis.

### 4.3 Positive symptom severity is correlated with immune function

Interestingly, we found that positive symptom severity was correlated with immune pathways, both in the full FEP cohort, and in the Scz subset. One explanation for these results is that psychosocial and environmental stressors, trigger innate immune pathways, for example via the Hypothalamic pituitary adrenal (HPA) axis. This has been reported in previous FEP studies (Di Nicola et al., 2013; Mondelli et al., 2011) and our results for positive symptoms are consistent with an upregulation in NF-κB signalling, which can be modulated by the above-mentioned stressors.

For the Scz subset we also found a correlation with multiple brain expressed categories, including glutamatergic synapses. Interestingly the glutamatergic signal was in part due to Neurogranin (NRGN), which was found to be downregulated in all groups, and this downregulation was significantly correlated with positive symptom severity in the Scz subset. NRGN was one of the first GWAS schizophrenia hits (Stefansson et al., 2009), and has been shown to be downregulated in brain tissue of schizophrenics (Broadbelt et al., 2006).

In contrast pathways correlated with Negative symptom severity were less interpretable. This was partially because we only identified 38 probes that were significantly correlated to negative symptoms, while we identified 203 for positive symptoms (see Supplementary Table 4). As a result, pathways for negative symptoms were almost exclusively driven by 8 ribosomal genes (RPL11, RPL21, RPL35, RPL4, RPL5, RPS3, RPS5, RPS6) (see Table 4). As such our results in the most general terms suggest that negative symptom severity is correlated with changes in translation.

### 4.4 Anti-psychotic medication reduces magnitude of psychosis blood signature

Our results suggest that the effect of antipsychotics reduces the detectable blood signature associated with psychosis, without introducing an overwhelmingly confounding gene expression signature specific to anti-psychotics. This is consistent with previous findings by Crespo-Facorro et al. (2015), where antipsychotics appeared to shift expression levels towards what is seen in HC. This reduces the magnitude of a medication-naive psychosis blood signature and introduces a confounding in the overall signature by introducing medication specific changes. It should be noted that 8 antipsychotic medications were used to stabilise patients in this study, and these are likely to have different non-specific effects on gene expression. This is expected to reduce the confounding effect specific to a single drug in the overall FEP - HC results. Moreover, FEP were recruited shortly after their first contact with Mental Health services and therefore were on average within the first two weeks of pharmacological treatment.

### 4.5 Limitation

A major limitation of this study is that we were unable to control for several important confounders such as BMI, smoking, drugs and medications other than antipsychotics. It is possible that these factors could have a significant impact on cell composition and gene expression in whole blood. While we did use methods such as surrogate variable analysis to detect potential major unknown confounders, and CellMix to estimate variations in cell composition, it is ultimately not possible to adjust for all possible sources of variation.

### 4.6 Conclusion

Overall, we found evidence for multiple disrupted pathways in the blood of first episode psychosis patients, specifically in immune signalling and transcription/translation. In addition, we found that positive symptom severity correlated with genes involved in immune signalling while negative symptom severity correlated with changes in transcription. These signatures were more pronounced in individuals meeting the criteria for a diagnosis of schizophrenia, but they were detectable to some extent in other types of psychosis. These effects could not be attributed to anti-psychotic medication administered to stabilise patients, since anti-psychotic free patients showed higher magnitude changes in gene expression.

The following are the supplementary data related to this article.

Supplementary Table 1 Clinical Demographics. Supplementary Table 1

Supplementary Table 2 Full differential expression results. Supplementary Table 2

Supplementary Table 3 Full results of enriched pathways. Supplementary Table 3

Supplementary Table 4 Full list of PANNS correlated genes and pathways. Supplementary Table 4

Supplementary Table 5 Full list of differentially expressed genes between medication groups. Supplementary Table 5

## Contributors

Authors DJL, SJN, RJD, COI, RMM and MDF contributed to the study design.

MDF, VM, PD, CP, KA and RMM oversaw the recruitment and collection of patient samples and data. SHL, CJC and GB generated Microarray data, HP and SJN performed initial processing of raw Microarray data. EC, SF, MC, DQ, OA, JL and COI collected and processed clinical and demographic data.

DJL performed bioinformatic analysis and wrote the manuscript. All authors contributed to and have approved the final manuscript.

## Funding sources

This work was supported by the NIHR Biomedical Research Centre for Mental Health and Biomedical Research Unit for Dementia at the South London and Maudsley NHS Foundation Trust and Kings College London; the King's Bioscience Institute (DJL); the Guy's and St Thomas' Charity Prize PhD Programme in Biomedical and Translational Science (DJL) and the NARSAD Young Investigator Award Grant (Grant number: 22604) (COI). KJA holds an Alberta Centennial Addiction and Mental Health Research Chair, funded by the Government of Alberta. MDF is funded by a Clinician Scientist Medical Research Council (MRC) fellowship. The GAP study was funded by the Maudsley Charitable fund and the BRC.

## Declaration of Competing Interest

The authors declare no conflict of interest.
